# A Bearing Fault Diagnosis Method Based on Wavelet Packet Transform and Convolutional Neural Network Optimized by Simulated Annealing Algorithm

**DOI:** 10.3390/s22041410

**Published:** 2022-02-12

**Authors:** Feng He, Qing Ye

**Affiliations:** School of Computer Science, Yangtze University, Jingzhou 430023, China; 201904596@yangtzeu.edu.cn

**Keywords:** simulated annealing, wavelet packet transform, convolutional neural network

## Abstract

Bearings are widely used in various electrical and mechanical equipment. As their core components, failures often have serious consequences. At present, most parameter adjustment methods are still manual adjustments of parameters. This adjustment method is easily affected by prior knowledge, easily falls into the local optimal solution, cannot obtain the global optimal solution, and requires a lot of resources. Therefore, this paper proposes a new method for bearing fault diagnosis based on wavelet packet transform and convolutional neural network optimized by a simulated annealing algorithm. Firstly, the original bearing vibration signal is extracted by wavelet packet transform to obtain the spectrogram, and then the obtained spectrogram is sent to the convolutional neural network for parameter adjustment, and finally the simulated annealing algorithm is used to adjust the parameters. To verify the effectiveness of the method, the bearing database of Case Western Reserve University is used for testing, and the traditional intelligent bearing fault diagnosis methods are compared. The results show that the new method for bearing fault diagnosis proposed in this paper has a better and more reliable diagnosis effect than the existing machine learning and deep learning methods.

## 1. Introduction

Bearings are a widely used, significant part of modern machinery and equipment. Bearing failure leads to significant failure time, increases maintenance costs, and may even reduce productivity [1]. Rolling element bearings are important mechanical parts of rotating machinery. They are also the main causes of basic industrial equipment failures, such as those of roll mills in steel mills, paper mills, and wind-turbine power plants, accounting for 51% of all failures [2]. Therefore, efficient and accurate bearing fault diagnosis methods play an important role in ensuring the function of the entire mechanical system, and the research on these methods is thus of great significance.

The study of bearing failure requires a large amount of original data, most of which are related to the state of motion. The state of motion can be reflected through vibration, sound, heat, electricity, etc. To check the health of a motor comprehensively, a state monitoring system is used to collect real-time data on the motor; after the motor has been running for a while, a large amount of data can be obtained [3]. However, these data are often processed with low efficiency in traditional bearing fault diagnosis methods, so a more efficient and reliable method is required.

Supported by a large amount of data, the traditional method of bearing fault diagnosis, which is based on manual experience, has gradually transitioned to data-driven, intelligent bearing fault diagnosis. Intelligent fault diagnosis can quickly and efficiently process the collected signals and provide accurate diagnoses. Thus, it is a promising tool in mechanical big-data processing. However, traditional intelligent diagnosis methods rely on prior knowledge and expertise for the manual extraction of features. These processes utilize human ingenuity, but they are time-consuming and labor-intensive [4].

One study [5] proposed a bearing fault diagnosis method combining the fuzzy c-means (FCM) method and an optimized k-nearest neighbor (KNN) model. Another [6] proposed a method to detect motor bearing faults. This method is based on spectral kurtosis (SK) and cross-correlation, extracts fault features representing different faults, and then uses principal component analysis (PCA) and semi-supervised KNN distance measure combines these features into a health index. Another study [7] proposed a sequential KNN classification method based on the affinity of distance and density to perform classification resulting in bearing fault diagnosis. In other study, a diagnosis method was proposed based on data-driven random fuzzy evidence collection and Dempster–Shafer evidence theory.

The literature [8] proposed a sequential KNN classification method based on the affinity of distance and density to perform classification for bearing fault diagnosis. The literature [9] proposed a diagnosis method based on data-driven random fuzzy evidence collection and Dempster–Shafer evidence theory. Then, by studying the bearing fault diagnosis method at this stage, it can be found that the bearing fault diagnosis method at this stage has obvious defects in feature extraction. In response to this problem, some scholars have proposed a new bearing fault diagnosis model, including feature extraction methods and fault classification methods.

Deep learning, which can discover features from raw data through multi-layer non-linear data-processing units, has become a promising tool for intelligent bearing fault diagnosis. Deep learning has a wide range of applications, such as pedestrian re-identification [10] and face alignment [11]. Indeed, deep learning has recently played a crucial role in the field of artificial intelligence [12]. Deep convolutional neural networks have strong data-mining and information integration capabilities, and they have been widely used in research on monitoring and diagnosing rotating machinery [13]. One study [14] proposed a method for feature extraction using continuous wavelet transform and rolling bearing fault diagnosis based on a convolutional neural network and support vector machine. Another study [15] proposed a bearing fault diagnosis method based on short-time Fourier transform and a convolutional neural network. Other studies have proposed bearing fault diagnosis methods based on the Stacked Inverted Residual Convolutional Neural Network (SIRCNN) and on the deep structure of the convolutional neural network [16,17]. Another novel fault diagnosis strategy was based on Synchronous Compression Transformation (SST) and deep convolutional neural network [16]. Someone proposed a bearing diagnosis method based on deep feature learning, in order to solve the fault identification of metal bearings, ceramic bearings, and hybrid bearings in electromechanical systems [18]. Some scholars have also proposed a bearing fault diagnosis method based on CNN. Compared with the traditional method, because CNN is based on the characteristics of weight sharing and sparse connection, it has a strong feature extraction ability, so it has a better effect in bearing fault diagnosis.

Although the bearing fault diagnosis based on convolutional neural network has achieved good results [19,20,21], two main problems remain. On the one hand, the training of convolutional neural networks is still a difficult problem. The training process often consumes a long time and requires more computing resources. In addition, it is easy to fall into the local optimal solution during the training process, resulting in a poor final recognition effect. Poor, based on the above problems, it will make the final industrial landing very difficult, and a lot of resources are spent in the adjustment process of parameters, but the setting of these parameters often has a decisive impact on the final result. On the other hand, when the parameters are adjusted, the convergence speed is too slow, and the local optimal solution is trapped, and it is difficult to find the global optimal solution. The above problems are factors that have a great influence on the bearing fault diagnosis effect, and need to be solved urgently.

Heuristic algorithms have a wide range of applications. In dealing with optimization problems, many excellent optimization algorithms have been proposed in recent years, such as flower pollination algorithms [22], generalized normal distribution algorithms based on local search [23], and improved genetic algorithms [24]. Some excellent heuristic algorithms have been proposed to solve optimization problems, which have achieved good results in various fields [25,26,27]. The heuristic algorithm mainly uses limited resources to obtain the optimal solution as much as possible in the case of limited resources, and in most cases, it can achieve good results. When training a convolutional neural network, time and computing resources are often limited. In the industry, when performing actual reasoning, it also needs to respond quickly, so it is suitable to use heuristic algorithms for optimization.

To address this problem, this study proposed a simulated annealing (SA) optimization method for bearing fault diagnosis based on a convolutional neural network and wavelet packet transform (WPT). Through WPT, the original signal was changed into a spectrogram. This spectrogram was then used as the input data of the convolutional neural network, which used feature-extracted samples for supervised training. Then, a SA algorithm was introduced to optimize the parameters of the convolutional neural network continually. The Case Western Reserve University bearing database was then used to verify this study’s proposed bearing fault diagnosis method.

This study also compared the proposed bearing fault diagnosis method with some traditional algorithms [28,29] including support vector machine, BP neural network, and WPT—convolutional neural network. Finally, the effectiveness of the proposed diagnosis method was verified through the comparison.

The innovation of this paper is as follows: a novel bearing fault diagnosis framework is proposed, based on a unique combination of the convolutional neural network, the wavelet packet transform and the simulated annealing algorithm.

This article is organized as follows: Section 2 introduces the data set used in the experiment, while Section 3 introduces the method proposed in this study, which included WPT, a convolutional neural network, and a SA algorithm. Section 4 describes the experiment and provides an analysis of the results. The Section 5 summarizes the article.

## 2. Data Set Description

**Figure 1:**
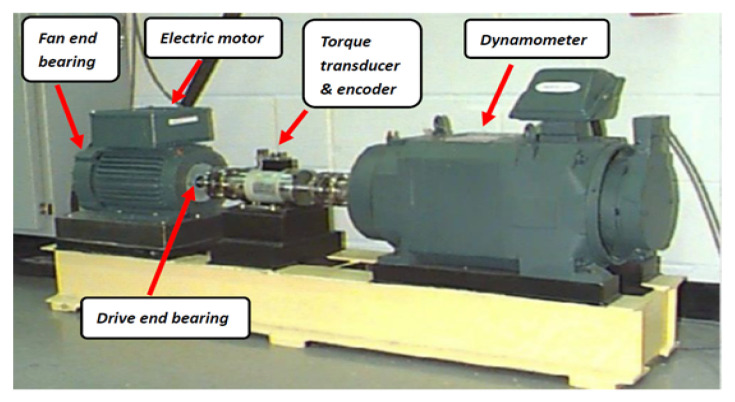
The experimental equipment.

The Case Western Reserve University bearing database was employed in this study because a large number of recent studies have been based on this data set and have achieved good results. The experimental equipment is shown in Figure 1. All bearings tested during the experiment were SKF bearings, which can be divided into three classifications: ordinary bearings, drive end (DE) bearings, and fan end (FE) bearings [30].

**Figure 2:**
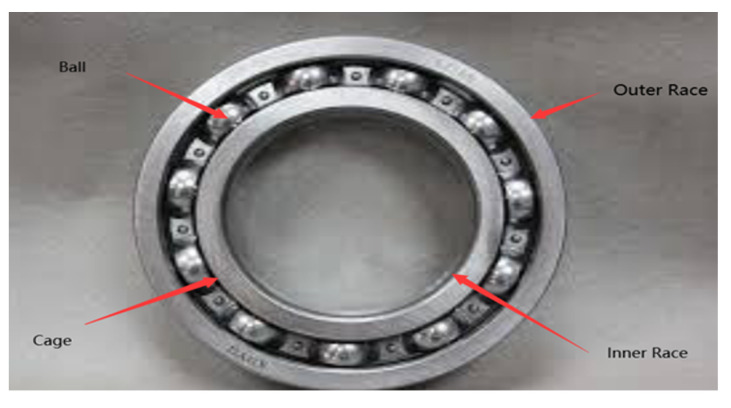
Bearing physical map.

The accelerometer installed on the drive end of the induction motor was used for data collection. The sampling frequency was 12 KHZ, and each vibration signal was 10 s. The collected data included four bearing states: (1) normal state, (2) inner ring failure, (3) rolling element failure, and (4) outer ring failure. Figure 2 provides a physical drawing of the bearing. The sampling was completed at different speeds to capture each of these states: 1750 rpm, 1772 rpm, and 1797 rpm. Empirical mode decomposition was used to introduce a single point of failure. The fault point size was 0.007, 0.014, 0.021, and 0.028 inches. The experiment was also repeated under different loads, including 0, 1, 2, and 3 horsepower (hp) [31].

The number of sampling points often has a great impact on the experimental results. Generally, as the number of sampling points increases, the final bearing effect will be correspondingly better. However, for application to the actual industrial field and realization of real-time bearing fault diagnosis, too many sampling points cannot be selected. To evaluate the effectiveness and robustness of the proposed method, 400 sampling points were selected per group. At present, most studies choose 1024, 2048, or 4096 for a single sampling point. A large number of sampling points will greatly improve the final diagnostic accuracy, but the real-time performance will also be affected. In subsequent experiments, the bearings were divided into 12 categories to verify the method proposed. The classification is shown in Table 1.

## 3. The Proposed Bearing Fault Diagnosis Method

This section introduces the bearing fault diagnosis method (SWC) based on a SA-optimized convolutional neural network and WPT. The widely used bearing fault diagnosis algorithm is mainly composed of feature extraction and pattern recognition. First, the main features of the original diagnostic signal are extracted. Then, a pattern recognition algorithm is used, and the extracted features are used as a data set for training, thereby achieving the purpose of bearing fault diagnosis. This methodology has been proven relatively mature and has achieved good results.

**Figure 3:**
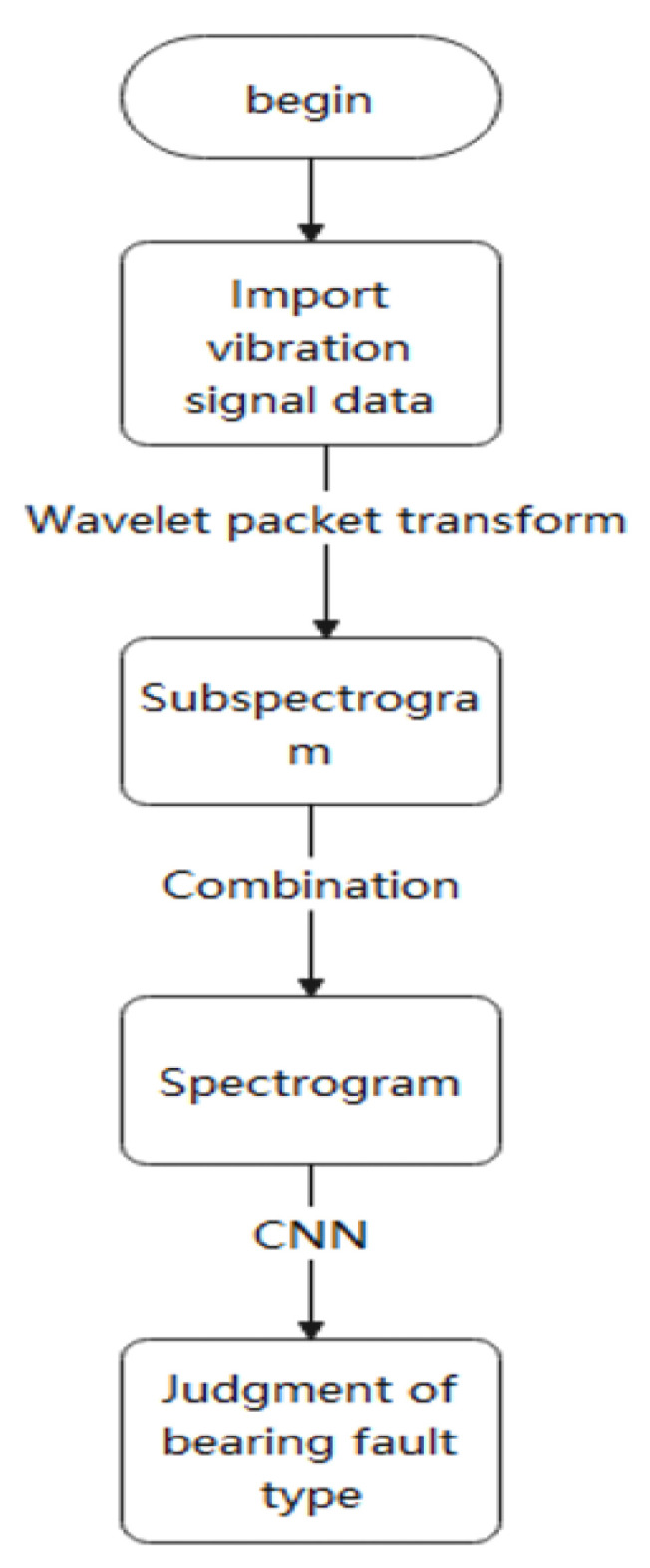
SWC algorithm flow chart.

**Figure 4:**
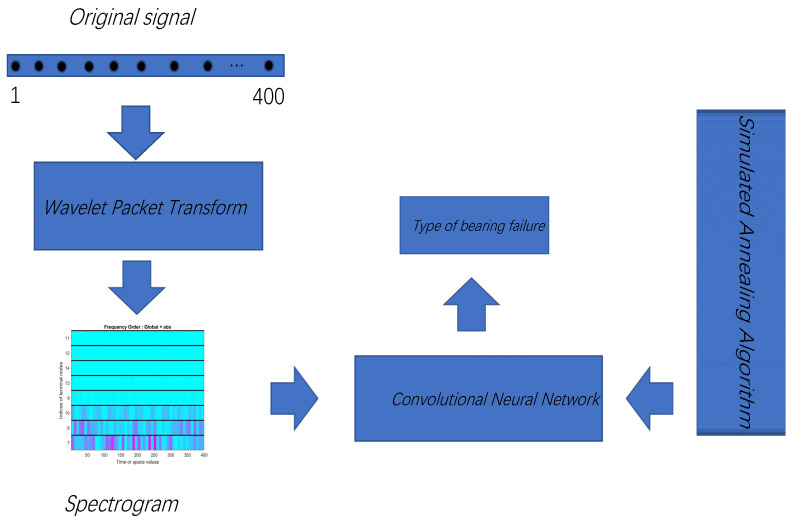
Schematic diagram of the method presented in this paper. First, input the original signal with a length of 400, perform wavelet packet transformation, and obtain a spectrogram. The obtained spectrogram is then used as a training data set and sent to the convolutional neural network. Finally, the simulated annealing algorithm is used to adjust the parameters of the convolutional neural network.

The proposed bearing fault diagnosis method (SWC) based on a convolutional neural network optimized by SA and WPT also roughly followed this model. On this basis, the SA algorithm was introduced for automatic parameter adjustment, which became a kind of self-adjustment and thus adapted the bearing fault diagnosis method. Figure 3 depicts the workflow of the SWC algorithm First, WPT was used for feature extraction to obtain a spectrogram. This spectrogram was then sent to the convolutional neural network for training. Finally, the SA algorithm was used to optimize the convolutional neural network, and the global optimal solution was obtained. Figure 4 briefly describes this process.

### 3.1. Feature Extraction Based on Wavelet Packet Transform

Machine-learning algorithms based on vibration signal processing are a common method used in bearing fault diagnosis [32]. Feature extraction of bearing signals is key for fault diagnosis, as the excellent features of bearing vibration signals aid in the accurate diagnosis of bearing faults. When a fault occurs, the time domain and the frequency domain of the output signal are different. WPT analysis can accurately find this fault information and location. Indeed, since WPT appeared in the early 1980s, its popularity has grown; WPT has many advantages over Fourier transform [33]. WPT is an important mathematic tool for analyzing nonlinear and non-stationary signals. It can decompose the signal into multiple sub-signals with different frequency ranges. Further, it can decompose the detailed information of the high-frequency area signal. Therefore, feature extraction of bearing vibration signals based on WPT is one of the most commonly used methods. The mathematical description of the WPT algorithm is as follows:

(1) $$W_{j,k}^{n}\left(t\right)=2^{\frac{j}{2}}W^{n}\left(2^{j}t-k\right).$$

where the integers *j* and *k* are the index scale and translation operations. The index *n* is an operation modulation parameter or oscillation parameter. In the process of WPT, the detected signal is divided by a pair of low-frequency and high-frequency filters into an approximate part and a detailed part, respectively. The approximate part is then further split into a secondary approximate part and a detailed part. Similarly, the first level of detail is split into a second level of approximation and detail. This process continues until the stopping criterion is reached. For n-level decomposition, the signal is decomposed into 2n narrowband signals. The recombined signal carries the same information as the original signal.

**Figure 5:**
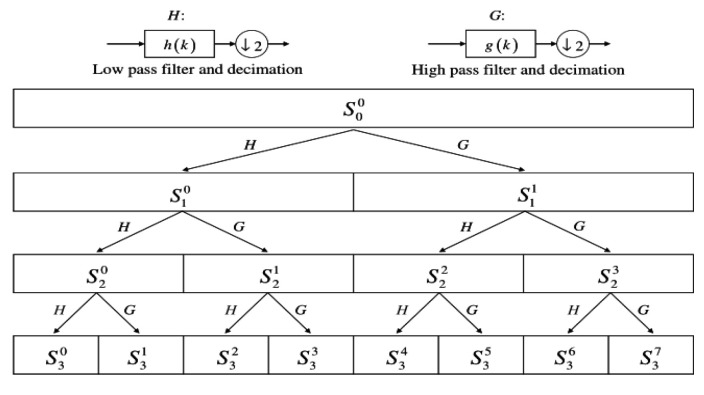
WPT decomposition tree structure [34].

In SWC, WPT was used as the signal extraction method. WPT had the same bandwidth in each decomposition, as shown in the decomposition tree structure provided in Figure 5. In this decomposition mode, the original signal would not increase or decrease, and thus it was retained to the greatest extent possible; almost no loss of the original signal occurred during the process. This facilitated the processing of non-stationary signals, and good time-frequency analysis could be performed, regardless of the high-frequency or low-frequency parts of the original signal.

**Figure 6:**
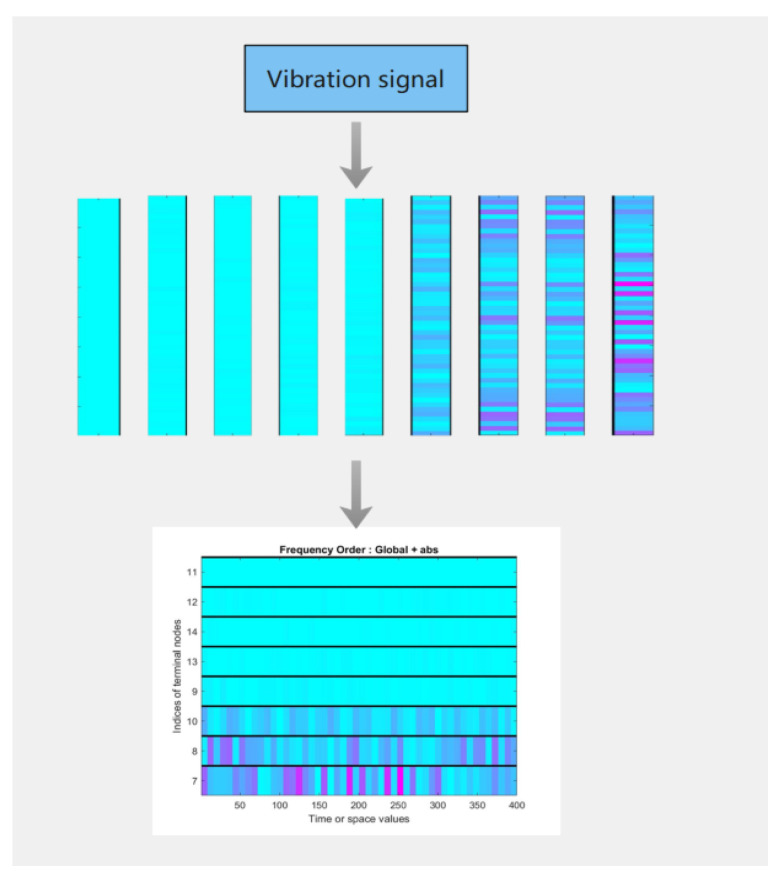
Schematic diagram of wavelet packet transform.

The Case Western Reserve University bearing database was used as a data set for analysis, and the approximate results are shown in Figure 6.

**Figure 7:**
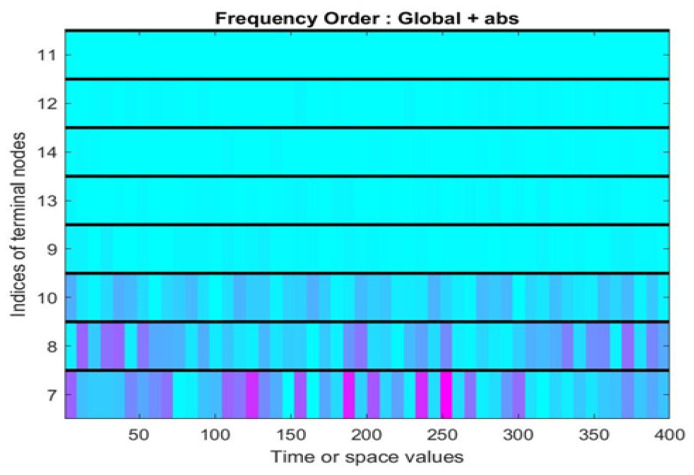
Timing signal spectrogram.

For timing signals, the characteristic frequency cannot be found directly Therefore, WPT was used to process the original timing signal and convert it to different frequencies, as shown in Figure 6. To improve the efficiency of data utilization, these pictures were spliced into one picture to use as the training data for the convolutional neural network, as shown in Figure 7.

### 3.2. Fault Classification Based on a Convolutional Neural Network

Deep learning has been widely used in various fields and has achieved relatively good results. Among deep learning algorithms, the development of convolutional neural network is the fastest [35]. With the continuous improvement of computer performance, the efficiency of convolutional neural networks in processing sound and video data sets far exceeds that of the traditional methods.

**Figure 8:**
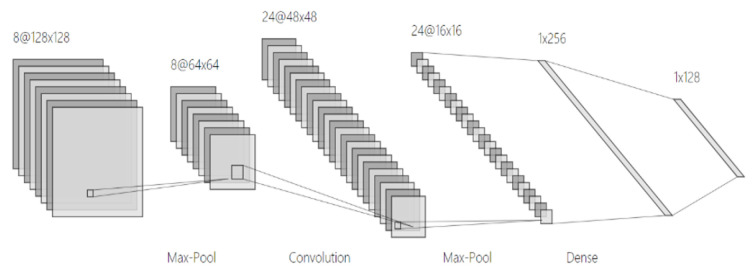
Convolutional neural network structure diagram.

Similarly, deep learning is also widely used in the field of bearing fault diagnosis. Compared with traditional diagnosis methods, bearing diagnosis based on convolutional neural networks has stronger anti-noise ability, faster processing speed, and higher accuracy. The structure of a convolutional neural network in shown in Figure 8.

#### 3.2.1. Convolutional Layer

The convolution layer of a convolutional neural network is mainly composed of several convolutional kernels, and the parameters of each kernel are optimized through a back propagation algorithm [36]. Convolution is a mathematical operation, and the completion of this operation in the convolution layer depends only on the convolution kernels. These kernels extract the features of the input data and perform learning.

The convolution layer can also complete local connection and weight sharing. Local connection means that the parameters of the convolution layer are only connectedwith the previous layer, and only local features are learned. This ensures that similar data will be input and similar answers will be obtained. Weight sharing reduces parameters and enables rapid convergence. Convolution can be described by the following mathematical formula:

(2) $$a_{i,j}=f\left(\sum_{m=0}^{2}\sum_{n=0}^{2}w_{m,n}x_{i+m,j+n}+w_{b}\right)$$

Among them, *f* represents the activation function, usually the relu function, which is described in detail below. *i* and *j* represent the element in the i-th row and j-th column of the input data matrix. *m* and *n* represent the weight of the *m*th row and *n* column, and $w_{b}$ represents the bias term.

#### 3.2.2. Activation Function

When the activation function is not used, each layer is a linear combination of the previous layer, so that fewer types of functions can be fitted. After introducing a non-linear activation function, the model can fit almost any non-linear function, namely. This activation function is introduced to improve the fitting ability of the model [14]. Activation functions are continuous and differentiable, which facilitates complex mathematical operations. Commonly used activation functions include sigmoid, relu, tanh, leaky relu, maxout, elu, etc.

The expression of the relu function is as follows:

(3) f(x)=max(0,x)

**Figure 9:**
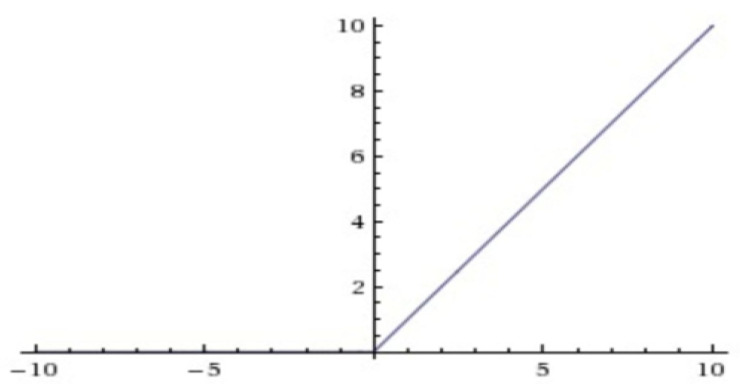
The relu function graph.

The image of the relu function is shown in Figure 9.

#### 3.2.3. Pooling Layer

**Figure 10:**
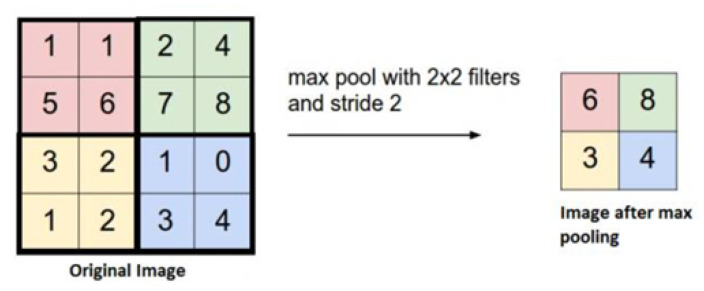
Schematic diagram of pooling process.

In simple terms, pooling is a non-linear down-sampling. The pooling layer completes two tasks. First, it reduces the input feature map to reduce the complexity of the model and optimize its calculation. Second, it compresses the features, extracts the main features, and reduces the phenomenon of over-fitting [37]. The process of pooling is roughly shown in Figure 10.

Common pooling includes maximum pooling and average pooling. The realization mechanism of maximum pooling is to divide the input matrix into multiple regions and select the maximum value for each subregion. Through this mechanism, the most important characteristics of the input data are extracted.

Some models no longer use the pooling layer, as pooling reduces the dimensionality of the data and simplifies the calculation. However, part of the original data is lost. This loss of the original data will cause part of the data to be lost, which is detrimental to the final effect of the model [38].

#### 3.2.4. Fully Connected Layer

Under normal circumstances, the data must be sent to the fully connected layer after convolutions and pooling to complete the final operation of the model. In the convolution layer, the local feature extraction of the input data is completed. If only the local features are used to judge, the phenomenon of over-fitting is prone to appear [39]. Therefore, all features must be used. Once the process is complete, the weight matrix reassembles the previously extracted features into a complete feature map.

#### 3.2.5. Fault Classification

In the proposed algorithm, the convolutional neural network algorithm is used for final pattern extraction to classify faults. Convolutional neural networks have many advantages in image recognition and classification. In this study’s comparative analysis, the classification effect of the convolutional neural network was compared with that of a support vector machine and BP neural network. The results showed that convolutional neural networks achieved better fault classification results.

The spectrogram was sent to the convolutional neural network for training. After training, the convolutional neural network calculated the fault type. As stated above, convolutional neural networks are composed of an input layer, convolution layer, pooling layer, and fully connected layer. The general structure is shown Figure.

**Figure 11:**
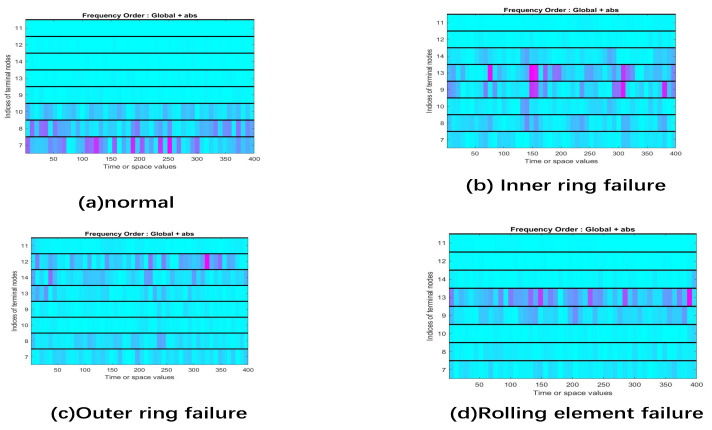
Comparison chart of four state bearings.

The state of bearings is classified as normal, inner-ring failure, outer-ring failure, and rolling element failure. Figure 11 shows the frequency spectrum of these four bearing states.

**Figure 12:**
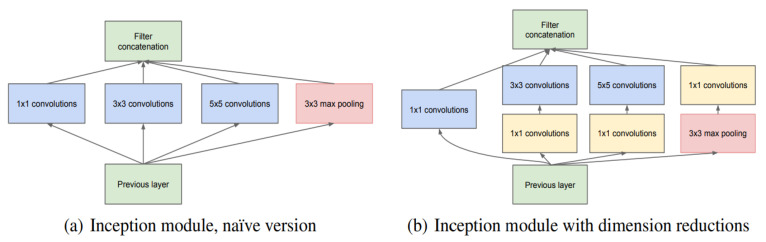
Convolutional neural network structure used in the experiment (partial) [25].

The signal-processed data were sent to the convolutional neural network as input data for training. The convolutional neural network of the present study is shown in Figure 12.

### 3.3. Optimization Based on Simulated Annealing Algorithm

The earliest idea of SA was proposed by N. Metropolis et al. in 1953. Then, in 1983, S. Kirkpatrick and others successfully introduced the idea of annealing to the field of combinatorial optimization, which is a stochastic optimization algorithm based on the Monte-Carlo iterative solution strategy similar to that used in [40]. The solution obtained by the SA algorithm has been shown to converge to the global optimal solution according to the probability theory.

**Figure 13:**
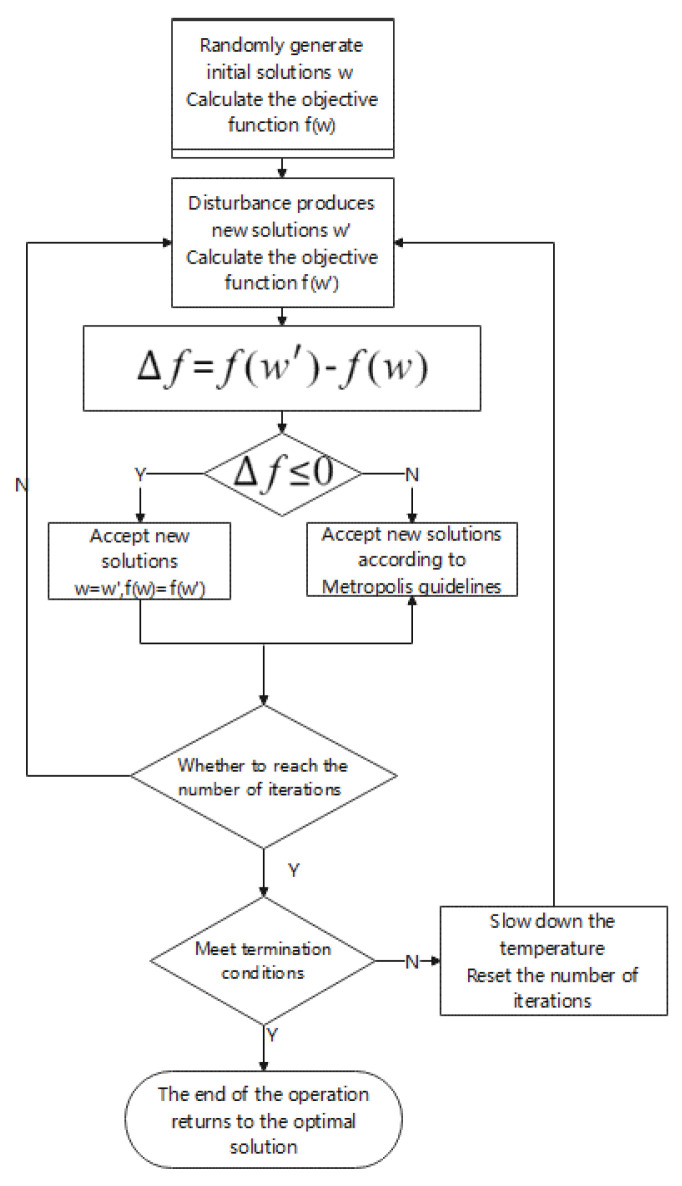
Flow chart of simulated annealing algorithm.

The core concept of SA can be roughly described as follows. As the initial annealing temperature drops, the optimal solution is constantly searched for within the feasible solution. Unlike the hill-climbing algorithm, the SA algorithm has a certain probability of accepting a solution that is worse than the original solution so that it can avoid getting trapped by local optimal solutions. The probability of accepting the poor solution continues to decrease until the SA finally approaches the global optimal solution. This process is just like that of metal annealing [41]. The general process is shown in Figure 13.

As a general random search algorithm, SA has been widely used in VLSI design, image recognition, and neural network computer research. However, this algorithm is not widely applied in the field of bearing fault diagnosis. Thus, the optimization potential of SA is obvious.

The parameter adjustment of the convolutional neural network is very important, as the quality of the parameter settings determines the performance of the final model. However, parameter adjustment is often difficult and requires a lot of manpower and computing resources. Additionally, because it is based on human experience, falling into the local optimal solution is easy, which renders obtaining the global optimal solution impossible. Therefore, the SA was introduced to improve the model. Due to the introduction of the SA algorithm, the final algorithm became an adaptive bearing fault diagnosis method. Compared with other diagnostic methods that require manual adjustment of parameters, this method had a better scope of application.

As an important optimization algorithm, the SA algorithm is mainly used to solve optimization problems in the engineering field. In this study, the SA algorithm was introduced to optimize the parameters of the convolutional neural network so that it could obtain a higher accuracy rate, reduce over-fitting, and accelerate the convergence speed. The setting of some parameters in the SA algorithm will affect the results of the final experiment. In the present study, the initial temperature was set to 1000 degrees Celsius, and the learning rate was 0.95.

In the previous section, the basic model architecture was described. WPT analysis was used as a signal processing method for feature extraction, and a convolutional neural network was used as a fault classification algorithm. Optimizing the convolutional neural network using a SA algorithm aimed to optimize the parameters of the convolutional neural network to help quickly find the most suitable parameters. This sped up the convergence of the convolutional neural network and obtained a higher accuracy rate.

The following steps were used to optimize the convolutional neural network with the SA algorithm:

Step 1: Specify the initial solution, set the initial temperature

Start by setting up an initial solution and use the initial default settings as the initial solution. The solution is treated as a set, including the required parameters for the learning rate and the number of iterations. Theoretically, the larger the initial temperature setting, the better, but since the higher the temperature, the time required for convergence will also increase accordingly, so it is necessary to make a compromise between the convergence time and the convergence accuracy, which is also the essence of the heuristic algorithm, where we choose 1000 degrees Celsius here, and choose 0.05 for the learning rate. With limited computational and time resources, such a setup can find the optimal solution faster.

Step 2: Given the objective function

As an optimization problem, a suitable optimization function needs to be given. Here, the accuracy of the final bearing fault diagnosis is selected as the objective function of the optimization problem. After many iterations of the simulated annealing algorithm, the value of the objective function is infinitely close to the global value. Optimal solution: The cost function is opposite to the objective function. Here, the cost function can be obtained by taking the inverse of the value of the objective function. The optimization goal here is to maximize the objective function and reduce the cost function as much as possible.

Step 3: Given the disturbance function

In order to help find the optimal solution quickly, a perturbation function needs to be given. The perturbation function jumps to 1–5% based on the original parameters. As a new solution: The perturbation function is the core of the modified algorithm. A new solution is generated by setting the perturbation function, and according to the new solution, the corresponding objective function value is generated. After the value is obtained, it is compared with the old objective function value.

Step 4: Given acceptance criteria

In this experiment, Metropolis criterion is used. For the new solution, it will be compared with the original solution first. If it is better than the original solution, it will choose to accept it. If the original solution is not superior, it will be accepted with a certain probability.

This idea of accepting inferior solutions will help jump out of the local optimal solution and provide the possibility to find the global optimal solution.

## 4. Analysis of Results

The proposed bearing fault diagnosis method based on WPT and a convolutional neural network optimized by the SA algorithm was implemented in MATLAB, and the method was completed on a machine with GTX1660ti GPU. The Case Western Reserve University bearing database was used for verification.

**Figure 14:**
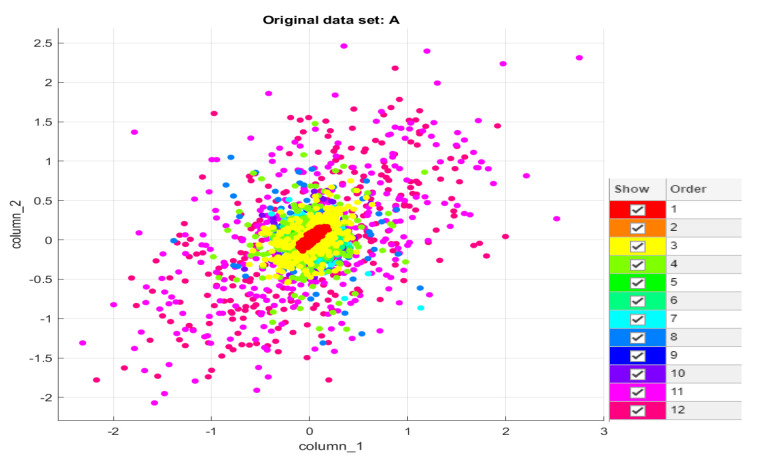
Visualization of test data.

Data containing 12 different types of bearing failures were used as test data. In Figure 14, we visualize the test data.

Bearing fault diagnosis consists of two processes: feature extraction and fault classification. The main feature extraction methods include principal component analysis, empirical mode decomposition, WPT, and short-time Fourier transform. In bearing fault diagnosis, the commonly used fault classification algorithms are BP neural network and support vector machine.

**Figure 15:**
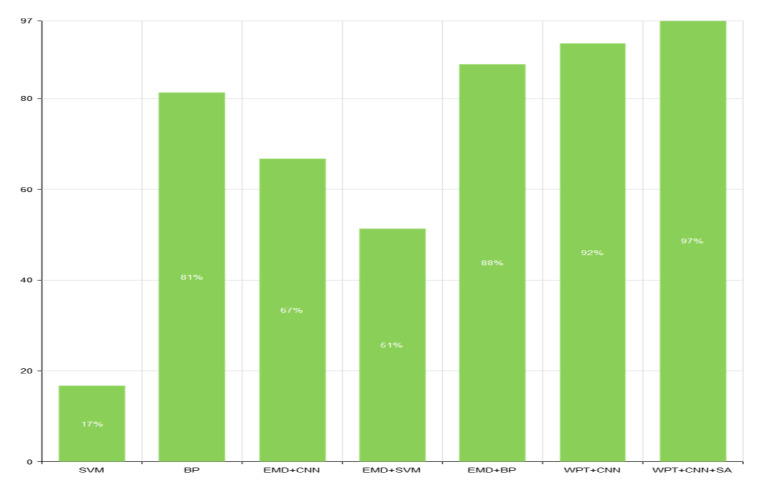
Comparative analysis of accuracy.

**Figure 16:**
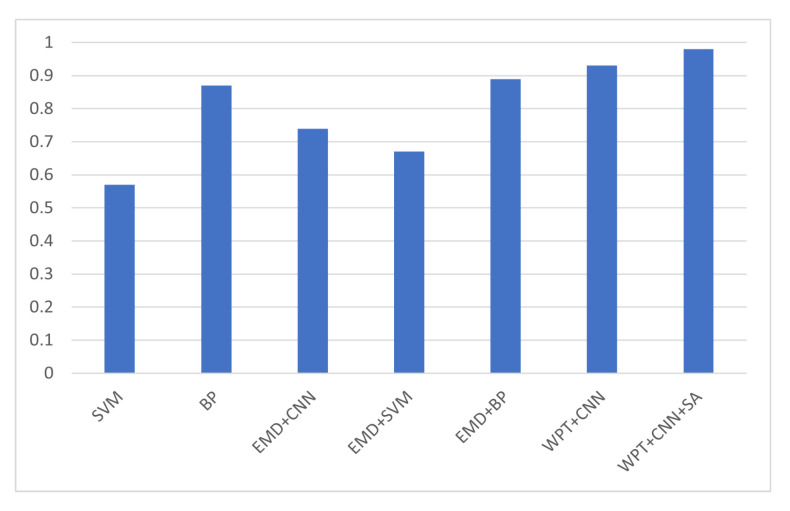
Comparison of AUC of different bearing fault diagnosis methods.

To illustrate the effectiveness of the proposed method, it was compared with the traditional intelligent algorithms. Compared with these algorithms, the results of the new bearing fault diagnosis method showed a higher accuracy rate, thus supporting the effectiveness of the algorithm. The results of the comparison are shown in Table 2, while Figure 15 provides a comparison of the traditional intelligent algorithms. In Figure 16, we compare the auc of different bearing fault diagnosis methods. The results show that the method proposed in this paper has the best classification effect among all the compared methods.

Empirical mode decomposition is an adaptive method that can decompose any signal into an empirical mode. It is commonly used for feature extraction in traditional intelligent bearing fault diagnosis algorithms. The empirical patterns obtained by re-composition are different oscillation patterns extracted from the original signal. Thus, the empirical mode can be used to reconstruct the original signal [42].

After analysis, the reason for obtaining better results is that, first, we use the wavelet packet transform to extract the features of the original signal to obtain the spectrogram, and then use the spectrogram as the training data set. Convolutional neural network is a good feature extractor, and after the original data has been subjected to wavelet packet transformation, it can obtain better diagnostic results. However, this still cannot achieve good results. Therefore, it is necessary to use a parameter adjustment method for the convolutional neural network. If the method of manually adjusting the parameters is used, it will consume a lot of time and computing resources. Heuristic algorithms can achieve better results when dealing with resource-constrained optimization problems. Therefore, the simulated annealing algorithm is introduced to adjust the parameters to obtain a better bearing fault diagnosis effect.

## 5. Discussion

This study proposed a new bearing fault diagnosis method based on WPT and a convolutional neural network optimized by a SA algorithm. Compared with traditional methods, the model completed the task of bearing fault diagnosis more accurately and intelligently.

The method used WPT to preprocess the original vibration data and generates a spectrogram, which was used as the training data for the convolutional neural network. Then, the simulated degradation algorithm was used to find the optimal parameters, thereby replacing the process of manual parameter adjustment. Finally, the public data set from Case Western Reserve University was used to verify the model, which showed good results.

This method was also compared with support vector machines and BP neural networks, along with other models that have not been optimized by SA algorithms. The final results showed that the proposed method had simpler parameter setting and a higher accuracy rate.

The volume of the convolutional neural network was relatively small, which is convenient for transplantation in the industrial field. Compared with large-scale networks, it more easily achieves cross-platform transplantation while ensuring a certain accuracy rate.

In addition, through comparative experiments, the superiority of our proposed method compared with previous methods is demonstrated, and the heuristic algorithm is also effective in the field of bearing fault diagnosis.

## 6. Conclusions

This paper proposes a new bearing fault diagnosis algorithm-SWT. Firstly, the wavelet packet transform is used for simple signal processing, and then the convolutional neural network is used for feature extraction and fault identification. Finally, to improve the speed of model convergence and the accuracy of diagnosis, a simulated annealing algorithm is used to adjust parameters. SWT can directly process the original signal without any manual feature extraction, which reduces the waste of human resources. The effectiveness of the proposed method is further demonstrated by comparative experiments. In the academic world, some new methods have been proposed recently, such as compression and acceleration of neural networks, and multi-modal bearing fault diagnosis methods. From the literature, they have achieved good results in many fields, but in bearing faults the field of diagnosis is less used. Therefore, in future work, we will try to combine the acceleration and compression of neural networks, multi-modality, and other new methods, in order to provide new research ideas for the field of bearing fault diagnosis.

## Figures and Tables

**Table 1:** Diagram of the experimental equipment.

Class	Name	Samples	Distribution	Description
1	normal_FE_1	846	22.2%	normal, 1 hp load
2	normal_DE_2	846	22.2%	normal, 2 hp load
3	Inner_FE_1_007	212	5.56%	0.007’’, 1 hp load
4	Inner_DE_1_007	212	5.56%	0.007’’, 1 hp load
5	Ball_FE_1_007	211	5.53%	0.007’’, 1 hp load
6	Ball_DE_1_007	211	5.53%	0.007’’, 1 hp load
7	Outer_FE_1_007	212	5.56%	0.007’’, 1 hp load
8	Outer_DE_1_007	212	5.56%	0.007’’, 1 hp load
9	Inner_FE_1_014	212	5.56%	0.014’’, 1 hp load
10	Inner_FE_2_014	212	5.56%	0.014’’, 2 hp load
11	Inner_DE_2_028	212	5.56%	0.028’’, 2 hp load
12	Inner_DE_3_028	212	5.56%	0.028’’, 3 hp load

**Table 2:** Experimental omparison. The experimental results come from the implementation of the existing methods. On the machine equipped with GTX 1660TI, the matlab neural network toolbox is used to conduct multiple experiments, and the average value is obtained to obtain the result.

Class	Method	Sample Number	Accuracy(%)
1	SVM	1000	16.67
2	BP	1000	81.31
3	EMD+CNN	1000	66.76
4	EMD+SVM	1000	51.32
5	EMD+BP	1000	87.53
6	WPT+CNN	1000	92.13
7	WPT+CNN+SA	1000	97.12
